# Reconstructing topography and extent of injury to the superior mesenteric artery plexus in right colectomy with extended D3 mesenterectomy: a composite multimodal 3-dimensional analysis

**DOI:** 10.1007/s00464-022-09200-2

**Published:** 2022-04-05

**Authors:** Javier A. Luzon, Yngve Thorsen, Liebert P. Nogueira, Solveig N. Andersen, Bjørn Edwin, Håvard J. Haugen, Dejan Ignjatovic, Bojan V. Stimec

**Affiliations:** 1 Institute of Clinical Medicine, Faculty of Medicine, University of Oslo, Oslo, Norway; 2 Division of Surgery, Department of Digestive Surgery, Akershus University Hospital, Lørenskog, Norway; 3 Department of Biomaterials, Institute of Clinical Dentistry, University of Oslo, Oslo, Norway; 4 Department of Pathology, Akershus University Hospital, Lørenskog, Norway; 5 The Intervention Centre, Oslo University Hospital, Oslo, Norway; 6 Anatomy Sector, Teaching Unit, Faculty of Medicine, University of Geneva, Geneva, Switzerland

**Keywords:** Superior mesenteric artery, Nerve plexus, Colon cancer, 3D imaging, 3D printing, Histology

## Abstract

**Background:**

Superior mesenteric artery plexus (SMAP) injury is reported to cause postoperative intractable diarrhea after pancreatic/colonic surgery with extended lymphadenectomy. This study aims to describe the SMAP microanatomy and extent of injury after right colectomy with extended D3 mesenterectomy for cancer.

**Methods:**

Three groups (I) anatomical dissection, (II) postmortem histology, and (III) surgical specimen histology were included. Nerve count and area were compared between groups II and III and paravascular sheath thickness between groups I and II. 3D models were generated through 3D histology, nanoCT scanning, and finally through 3D printing.

**Results:**

A total of 21 specimens were included as follows: Group (I): 5 (3 females, 80–93 years), the SMAP is a complex mesh surrounding the superior mesenteric artery (SMA), branching out, following peripheral arteries and intertwining between them, (II): 7 (5 females, 71–86 years), nerve count: 53 ± 12.42 (38–68), and area: 1.84 ± 0.50 mm^2^ (1.16–2.29), and (III): 9 (5 females, 55–69 years), nerve count: 31.6 ± 6.74 (range 23–43), and area: 0.889 ± 0.45 mm^2^ (range 0.479–1.668). SMAP transection injury is 59% of nerve count and 48% of nerve area at middle colic artery origin level. The median values of paravascular sheath thickness decreased caudally from 2.05 to 1.04 mm (anatomical dissection) and from 2.65 to 1.17 mm (postmortem histology). 3D histology models present nerve fibers exclusively within the paravascular sheath, and lymph nodes were observed only outside. NanoCT-derived models reveal oblique nerve fiber trajectories with inclinations between 35° and 55°. Two 3D-printed models of the SMAP were also achieved in a 1:2 scale.

**Conclusion:**

SMAP surrounds the SMA and branches within the paravascular sheath, while bowel lymph nodes and vessels lie outside. Extent of SMAP injury on histological slides (transection only) was 48% nerve area and 59% nerve count. The 35°–55° inclination range of SMAP nerves possibly imply an even larger injury when plexus excision is performed (lymphadenectomy). Reasons for later improvement of bowel function in these patients can lie in the interarterial nerve fibers between SMA branches.

What is known regarding the consequences of injury to the superior mesenteric artery plexus (SMAP) in humans is mostly derived from articles on pancreas cancer surgery and small bowel transplantation [1–6]. These procedures cause both intrinsic and extrinsic injuries to the bowel innervation, and this injury has been linked to “postoperative intractable diarrhea” [7, 8]. Such conclusions have further been transferred to right-sided colonic cancer surgery through analogy, despite the fact that only extrinsic injury to the SMAP can be induced here [9]. On the other hand, Gray’s anatomy describes the SMAP as paravascular postganglionic fibers that continue along the superior mesenteric artery (SMA) and its branches, without innervating these [10]. This description leaves room for further research on the subject.

A recent increase of the GI surgeons’ interest on postoperative bowel function after right colectomy for cancer has been noticed as complete mesocolic excision (CME) [11, 12], with extended mesenterectomy, is in the spotlight [13]. Publications imply a short period of diarrhea after surgery when injury to the SMAP is suspected [9, 14]. The main challenge is the quantification of the extent of injury to the plexus, something that will allow us to connect the symptoms with the cause.

The primary aim of this study is to establish the extent of injury to the SMAP while performing right colectomy with extended D3 mesenterectomy for cancer. A secondary aim is to present additional data on the macro- and microanatomy of the SMAP using composite methodology.

## Methods

Three different specimen groups underwent diverse methodologies of anatomical analysis. All specimen groups represented the same anatomical area—*The D3 volume* (level 3 of lymph node dissection) for the right colon, defined as mesenteric fat enclosing the superior mesenteric vessels between the following borders [15, 16]:

1. Cranial border: 5 mm from the origin of the middle colic artery (MCA).
2. Lateral border: 1 cm to the right from the superior mesenteric vein (SMV).
3. Caudal border: 1 cm distal to the line passing through the origins of the ileocolic vein (ICV) and ileocolic artery (ICA).
4. Medial border: the line along the left side of the SMA.

### Specimen acquisition

#### Group I: anatomical dissection specimens (formalin-fixed human cadavers)

These were harvested from bodies retrieved from University of Geneva willed body donation program. As no personal identifiers, apart from sex and age, were implied (anonymous biological material), an internal review board was not needed (Human Research Act 810.30, HRA).

**Fig. 1:**
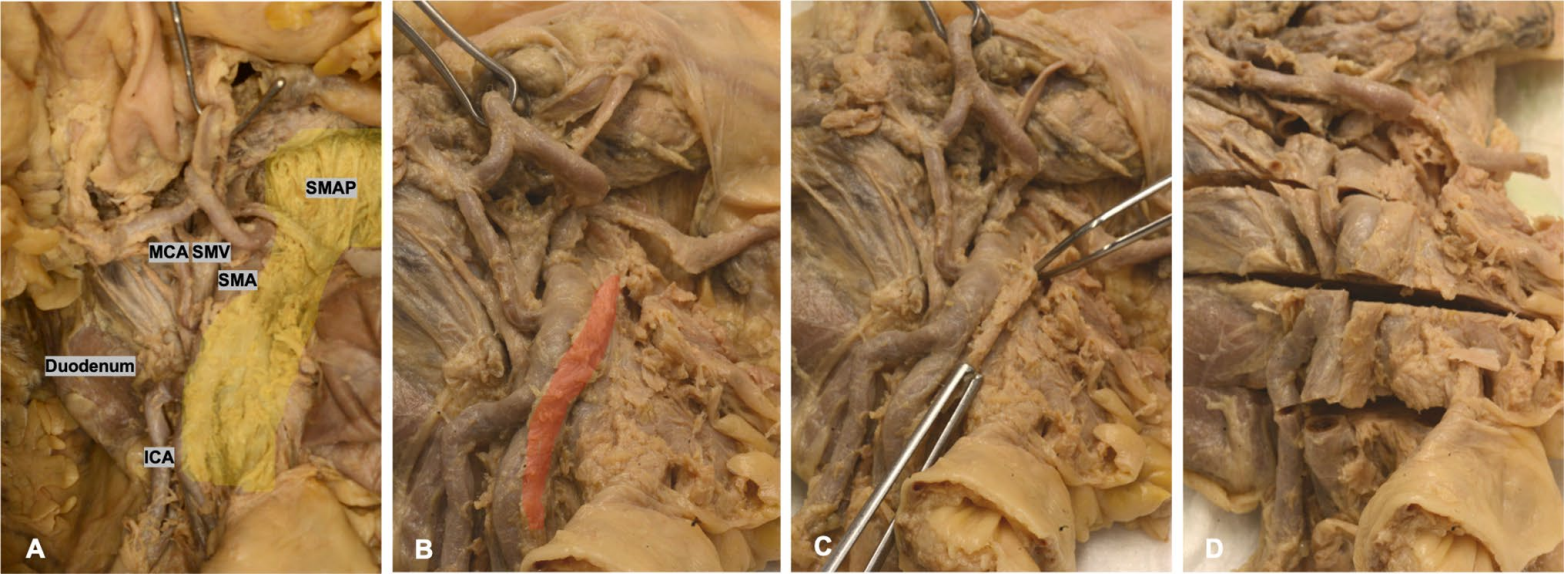
Stepwise dissection of the paravascular SMA sheath (Group I anatomical dissection). **A** Transparent yellow area highlights the trajectory of the SMAP along the SMA. **B** Transparent red area highlights the resected SMA sheath. **C** Surgical tweezers holding rests of the SMA sheath. **D** Specimen transection at 5 different levels for SMA sheath thickness measurement. *SMAP* superior mesenteric artery plexus, *SMV* superior mesenteric vein, *SMA* superior mesenteric artery, *MCA* middle colic artery, and *ICA* ileocolic artery

**Fig. 2:**
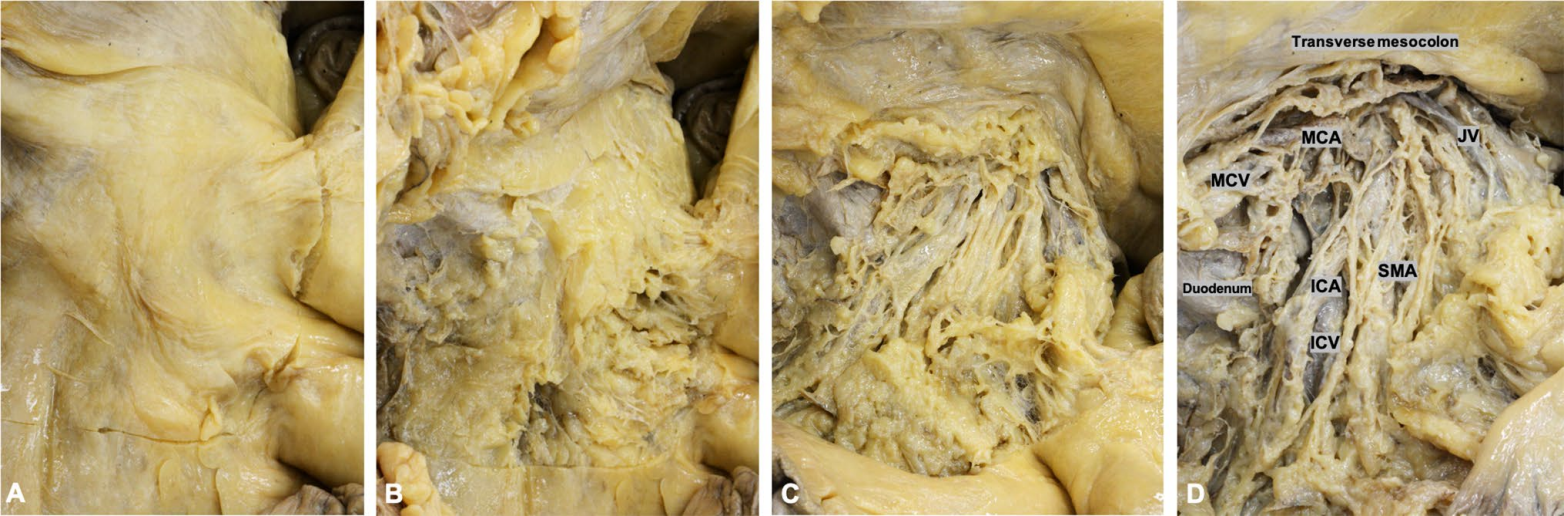
Stepwise dissection of exposing the SMAP (Group I anatomical dissection). **A** Dissection starts with initial incisions marking the limits of the D3 area. **B** Removal of peritoneum. **C** Subperitoneal fat/mesenteric tissue removed. **D** Finished dissection revealing vasculature and nerve fibers. *MCA* middle colic artery, *MCV* middle colic vein, *ICA* ileocolic artery, *ICV* ileocolic vein, *SMA* superior mesenteric artery, *JV* jejunal vein

The dissection followed the previously published description of extended D3 mesenterectomy [16]. The D3 volume was dissected in a stepwise stratigraphic manner, clearing the subperitoneal fat until further dissection allowed the identification of a layer of tissue between the surface of the SMA adventitia and the multiple collagenous and nerve fibers surrounding the periphery of the SMA, defined in this study as *the SMA sheath *[17] (Fig. 1). First, the intermediate and central lymph vessels and nodes were identified and cleared off to obtain a better insight in the branches of the SMA and affluents of the SMV. Second, the SMA sheath was followed throughout the proximal course of the MCA, RCA (if present), and ICA, as well as along the SMA left border and emergence of the jejunal arteries. If the jejunal veins were in the way, they were looped and outspread. The superior mesenteric artery plexus (SMAP) was carefully exposed and dissected free cranially to above the pancreatic notch (Fig. 2). Then, the sheath, together with its corresponding nerve fibers, was excised from the origin of the ileocolic artery (ICA) to the origin of the middle colic artery (MCA), leaving behind a nerve “stream” posterior to the SMA and to the left lateral side to the jejunal arteries. The dissected tissue was sampled for histological staining to confirm the presence of the nerve fibers within. The whole remaining specimen was subsequently serially sectioned in the axial plane, at five levels: 5 mm above the MCA origin, at the MCA origin, at between MCA-ICA origins, at the ICA origin, and 1 cm below the ICA origin (Fig. 1D). The thickness of the paravascular sheath was measured via a digital caliper (S Cal Work, Sylvac, CH).

#### Group II: postmortem D3 volume specimens (fresh human cadavers)

Postmortem D3 specimens were collected from cadavers during routine necropsy at the Department of Pathology at Akershus University Hospital from 2017 to 2018. Ethical approval *#2013/203 by the regional ethical committee*, (*REK Sør-Øst, Norway*). Family consent was secured.

Criteria for cadaver selection were as follows:

- No previous abdominal surgery or cancer diagnosis in the gastrointestinal system.
- An abdominal CT scan available prior to necropsy.
- Maximum of two days after the time of death.

The abdominal cavity was untouched before necropsy examination, in order for the surgeons to collect the specimen. Two colorectal surgeons (DI and YT) performed a laparotomy using the same technique as previously described to harvest the D3 volume [16]. For this group, the D3 specimen consisted of a block of mesenteric tissue, *including* the resected mesenteric vessels. Specimens were immediately placed into 100% ethanol for conservation purposes before further staining and imaging.

#### Group III: surgical D3 volume specimens

These were collected after elective surgery performed from 2013 to 2015 on patients included in the “Safe Radical D3 right hemicolectomy for cancer through preoperative biphasic multi-detector computed tomography (MDCT) angiography” clinical trial with registration at ClinicalTrials.gov (Identifier: NCT01351714) on May 9, 2011. *Ethical approval #*2010/3354 *by the regional ethical committee*, (*REK Sør-Øst, Norway*). Patients were required to sign an informed consent form before inclusion.

**Fig. 3:**
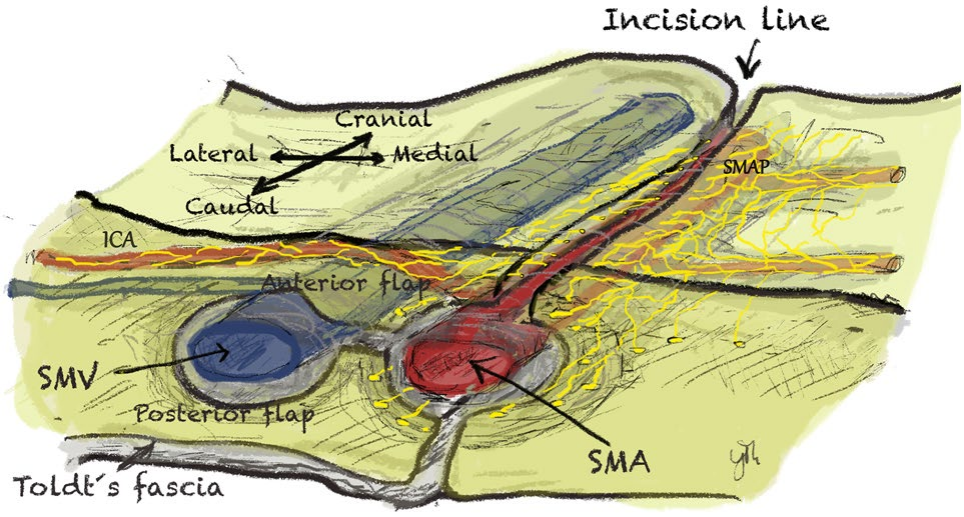
Surgical procedure (Group III surgical specimen): releasing the D3 volume along the left side of the SMA and removing both anterior and posterior flaps around the SMV, together with the paravascular sheath of the SMA. *SMAP* superior mesenteric artery plexus, *SMA* superior mesenteric artery, *SMV* superior mesenteric vein and *ICA* ileocolic artery

A transverse incision was made in the visceral peritoneum over the terminal ileal vessels 1 cm caudal to the origin of the ileocolic artery. After opening the vascular sheaths, the terminal ileal vessels were secured in vessel loops. The anterior flap of mesenteric tissue along the left side of the superior mesenteric artery (SMA) was progressively established and the surgical specimen was devascularized [ileocolic artery (ICA), right colic artery (RCA), and the middle colic artery (MCA) or its right branch were divided at their origin]. The surgical specimen was fully mobilized in order to provide access to the posterior flap of mesenteric tissue. The medial border of the posterior flap, behind the superior mesenteric vein (SMV) and SMA, was divided along the SMA. In this manner, all tissues (both anterior and posterior flaps) were removed en bloc with the specimen containing the central lymph vessels and nodes, the D3 volume (Fig. 3). In this study, D3 volumes underwent further histological sectioning as further explained below*.*

### Imaging techniques

The following imaging techniques were controlled by previously obtained CT-derived vascular reconstructions in order to secure correct anatomical orientation.

#### Destructive reconstruction (traditional histology slides and 3D histological models)

D3 volume specimens from groups II and III were fixed in 10% formaldehyde before cut in 4-mm-thick serial blocks and processed further to paraffin wax embedded tissue. These blocks were cut in 3–4 um thin sections at intervals of 50 um using a microtome and stained with hematoxylin phloxine saffron (HPS), before scanning using the Aperio ScanScope AT (Leica Biosystems) slide scanner with 20× magnification. Additionally, a selection of histological slides was performed for immunohistochemical analysis with the S-100 protein to identify nerve fibers. The immunohistochemical protocol has been described elsewhere [18].

##### Nerve count, diameter, and total area in traditional histological slides

These values were obtained with the annotation tool on the software Aperio ImageScope v.12.4.3 (Leica Biosystems) on histological slides. The *free-form* drawing tool was applied by coloring the borders of each nerve fiber where this action also automatically created a table with the number of structures selected along with its corresponding diameter in mm and area in mm^2^. Total nerve area (TNA) is defined as the sum of each nerve fiber area value at the level of the middle colic artery origin within the D3 area. SMA sheath thickness in Group II specimens was measured with the *digital ruler* tool in ImageScope software.

##### Alignment and volume reconstruction

Histological images were imported as stacks into a free-ware 3D histology reconstruction software HistoloZee v.0.3 [19], where each digital slide was aligned by *rotation, translation*, and *scaling* tools in HistoloZee to achieve a correct 3D reconstruction volume. Once alignment was secured at the histological level, *manual segmentation* tool on HistoloZee was used to delineate the walls of each nerve fiber per slide image. The same segmentation technique was performed in arteries, veins, and lymph nodes. All histological images were later exported to the open-source image processing software ITK-snap (www.itksnap.org) [20] in order to create a 3D model.

#### Nondestructive reconstruction (3D nanoCT models)

##### Specimen staining and preparation

Staining of selected Group II specimens was composed of two steps: *fixation* and *soft tissue staining*. Fixation was achieved by specimen submersion in absolute ethanol for 24 h. After fixation, the specimen is rinsed in 70% ethanol for 20 min. Phosphotungstic acid (PTA) powder was used to create a 0.7% ethanol-PTA (EPTA) solution for staining. The specimen is fully submerged in this *staining mixture* for 7 days and later preserved in absolute ethanol before scanning [21–24].

##### Nano-computer tomography (nanoCT) scanning and image reconstruction

The multiscale X-ray NanoCT-SkyScan 2211 (SkyScan, Bruker, Kontich, Belgium) is used to produce 3D models of Group II specimens. The acquisition parameters were set to 65 kV and 82 µA, exposure 120 ms with an averaging frame of 4 images. The whole dataset of projections was acquired at full rotation of 360° with a rotation step of 0.37°. An aluminum filter was used to remove the low-energy X-ray from the beam. Samples were scanned at a final isotropic pixel size of 35 um. After that, the images were reconstructed with NRecon software (v.1.7.4.1, Skyscan, Bruker, Belgium) using the modified Feldkamp cone-beam reconstruction algorithm, with the same parameters for all samples.

##### Image processing (segmentation) (ITK-snap)

Once the nanoCT image dataset was exported in DICOM format, images were imported into the ITK-snap. For nerve reconstruction, the thresholding function is applied where the PTA-enhanced nerves have a higher intensity than the surrounding structures. This model is later exported in STL format for further 3D visualization. Nerve fiber representations on the nanoCT 3D reconstructions were confirmed through direct comparison to the nerves identified on matching histological slides (at the same specimen level).

### 3D printing techniques

Two virtual 3D models (one derived after the nanoCT scanning and one from 3D histological reconstruction) were exported as STL files into the software Ultimaker Cura version 4.9.1, which is a 3D printing preparation interface. A layer size of 0.1 mm was chosen in this software as pre-printing settings. Next, the 3D printer Ultimaker model S3 (Ultimaker B.V. Utrecht, the Netherlands) was used to print the 3D models along with Ultimaker Pearl-White PLA (polylactic acid) filaments. Support structures were printed using the water-soluble filament Ultimaker PVA (polyvinyl alcohol).

Depending on the complexity of the model, the process took 24–32 h. These physical 3D models were printed at a 1:2 ratio from its original size, for illustrative purposes.

After the 3D printing process concluded, the models were left to cool down inside the 3D printer machine and later moved in a container with water for several hours for the dissolving of support structures.

### Statistical analysis

Descriptive statistics was performed in SPSS (IBM Inc., Chicago, IL, USA). Independent t test was performed to establish statistical significance in nerve analysis between Groups II and III.

## Results

The study included 21 specimens. Group I contained five specimens (3 females; age 80–93 years), which underwent anatomical dissection. Group II contained seven specimens (5 females, age 71–86 years), where two were randomly selected to first undergo NanoCT scanning. All specimens ultimately underwent serial histological sectioning. Another two specimens were selected for 3D histology reconstruction. Group III contained nine postoperative specimens (5 females, age 55–69 years), where all directly underwent serial histological sectioning.

Dimensions of Group II specimens had a mean of 5.83 cm (± SD 0.76) in length and 2.57 cm (± SD 0.51) in width. The dimensions of Group III specimens had a mean of 6.48 cm in length (± SD 2.07) and 2.65 cm (± SD 0.54) in width.

### Group I anatomical dissection

**Fig. 4:**
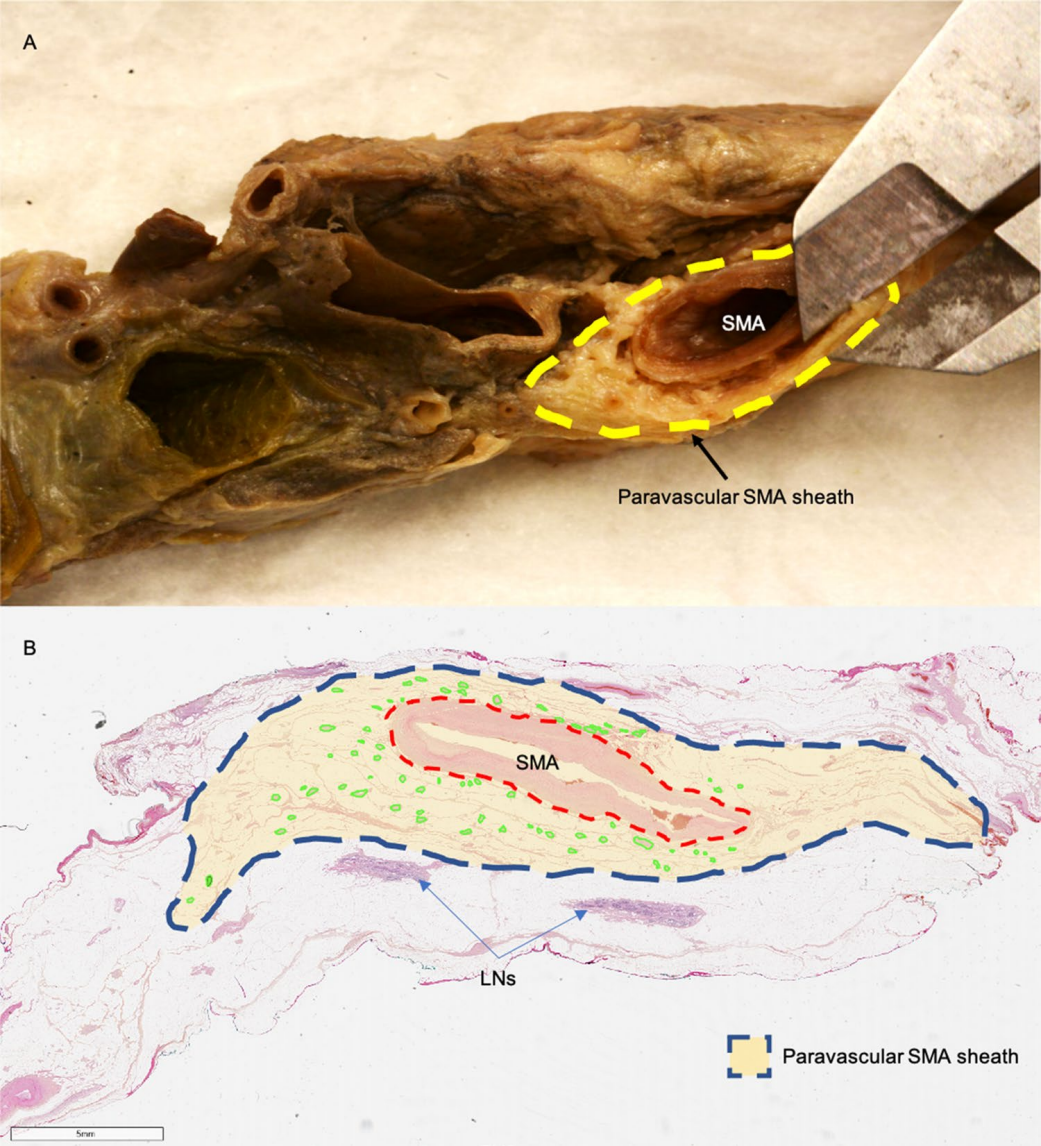
The paravascular superior mesenteric artery sheath. **A** SMA sheath thickness measurement on Group I anatomical dissection specimen with a digital caliper. **B** Histological slide from Group II postmortem specimen. Transparent yellow area represents the paravascular SMA sheath. Red-dotted line represents the outer border of the SMA adventitia. Green circles represent SMA plexus nerve fibers. *LNs* lymph nodes, *SMA* superior mesenteric artery (Color figure online)

A single layer, allowing for vascular dissection, located between the surface of SMA adventitia and the multiple collagenous layers at the periphery of the SMA was found and identified as the *vascular sheath of the SMA*. This area contains collagenous fibers, adipose tissue, lymph ducts, and multiple nerve fibers encircling the SMA (the SMA plexus). This plexus appeared as a mesh-like structure consisting of a complex network of nerve fibers. Larger parts of the central network of the SMA plexus lie anteriorly to the SMA, while a smaller number of fibers lie on the posterior and bilateral areas to the SMA (Fig. 4). These nerves do not innervate the SMA itself. Group I specimen dissections allowed distinguishing the network of the SMA plexus in two parts: a central network, which surrounds the main trunk of the SMA and a peripheral network located outside the D3 volume which belongs to the nerve fibers of the peripheral arterial branches and are observed as individual nerve threads that run along small bowel arteries. These nerve branches not only intertwine with each other but also branch off to the so-called “inter-vessel” or “non-vessel” spaces (mesenteric spaces of arterial loops), whereby their links are distributed transversely to the course of small bowel arteries.

### Group II histological nerve analysis

**Fig. 5:**
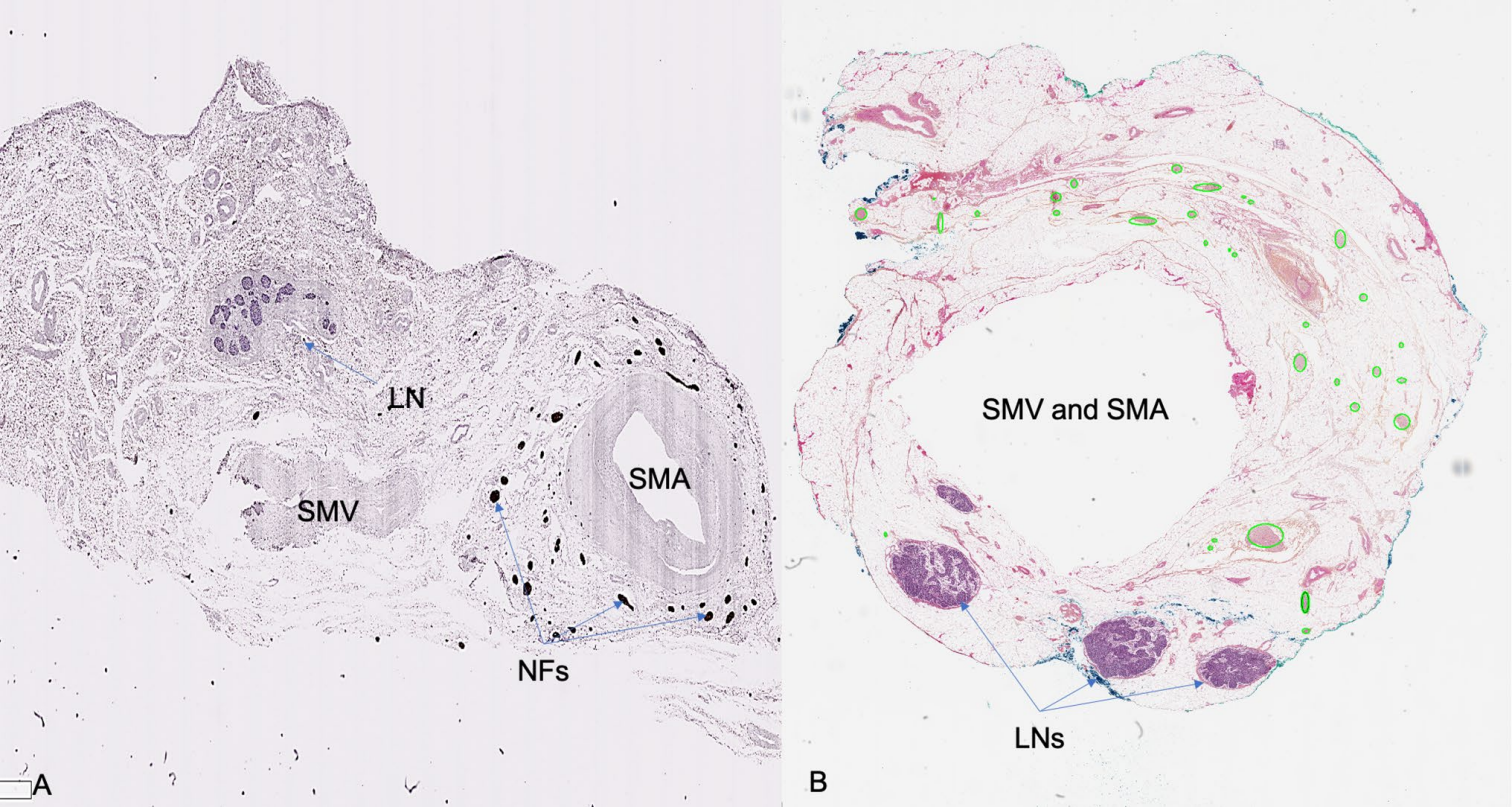
Histological specimens. **A** Group II postmortem specimens with S-100 protein staining specific for nerve fibers (NFs). **B** Group III surgical specimens with HPS staining. Nerve fibers marked in green are removed at surgery. Blank area in the middle represents the original location of the SMA and SMV. Green delineation represents anterior side and blue delineation represents posterior side for specimen orientation (Color figure online)

The mean *nerve count* was 53, with a median of 54 nerves (range 38–68, ± SD 12.42) among all specimens. The mean *nerve diameter* was 648.82 um with a median of 636.20 um (range 537.88–798.60, ± SD 79.17) among all specimens. Mean value of the *total nerve area* (TNA) was 1.84 mm^2^. The median value was 2.07 mm^2^ (range 1.16–2.29, ± SD 0.50). Group II histological slides showed no lymph nodes within the SMA sheath (Fig. 5A).

### Group III histological nerve analysis

The mean *nerve count* was 31.6 among all specimens, with a median of 30 (range 23–43, ± SD 6.74). The mean *nerve diameter* was 504.29 um with a median of 490.81 um (range 417.08–597.65, ± SD 66.52). Mean value of the *total nerve area* (TNA) was 0.889 mm^2^, with a median of 0.754 mm^2^ (range 0.479–1.668 ± SD 0.45). (Fig. 5B).

### Comparative analysis

Median values of SMA paravascular sheath thickness in Group I were 2.05 mm (range 1.67–2.69), 1.89 mm (1.55–2.26), 1.68 mm (1.03–2.11), 1.23 mm (0.77–1.83), and 1.04 mm (0.51–1.46) and in Group II 2.65 mm (range 2.06–3.20), 2.51 mm (1.99–3.01), 2.32 mm (1.75–2.90), 2.14 mm (1.48–2.66), and 1.17 mm (0.68–1.85), at the following specimen levels for both groups: 5 mm above MCA origin, at MCA origin, between MCA-ICA origins, at ICA origin, and 1 cm below ICA origin, respectively. Histologically, mean nerve count ratio between group II and group III specimens was 0.59, indicating 59% of nerve fibers were harvested in group III specimens and 41% of nerve fibers remained in the patient. Mean TNA difference between groups II and group III showed a ratio of 0.48. Thus, indicating that 48% of TNA was harvested in group III specimens, while 52% of TNA remained in the patient. There was a statistically significant difference in nerve number, nerve diameter, and TNA between groups II and III specimens, *p* = 0.0003, *p* = 0.0006, *p* = 0.0007, respectively).

### 3D models result

**Fig. 6:**
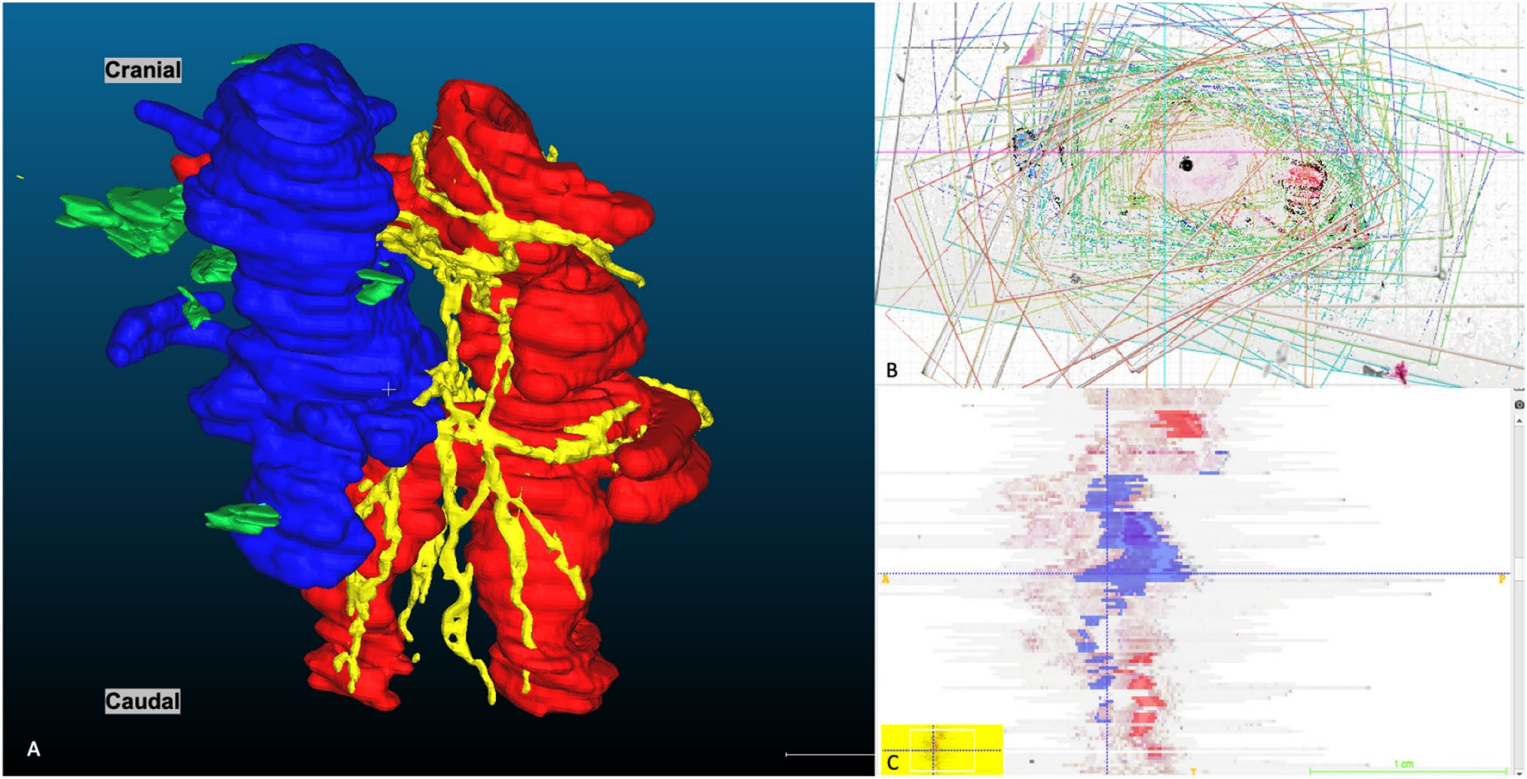
*Destructive* 3D histological reconstruction (Group II postmortem specimens). **A** Superior mesenteric artery and vein (red and blue, respectively). Green structures represent lymph nodes and yellow structures represent nerve fibers of the SMA plexus. Only nerves between the range of 300 and 800 um in diameter were reconstructed and portrayed in this 3D model. **B** Axial view of histology stack on HistoloZee. **C** Coronal view of histology stack with structures labeling (Color figure online)

**Fig. 7:**
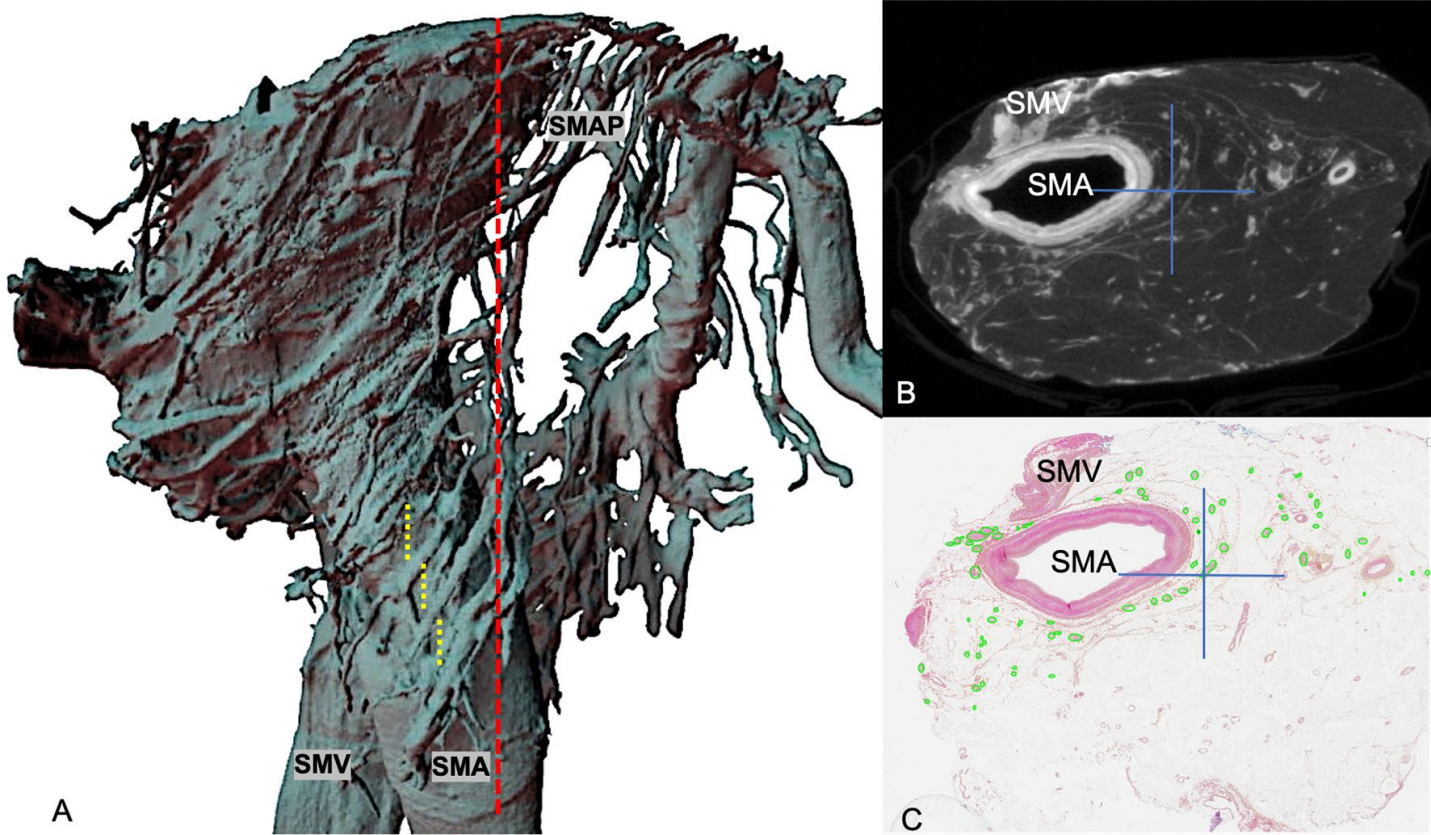
*Nondestructive* nanoCT 3D reconstruction (Group II postmortem specimens). **A** Dotted red line represents excision line at surgery. Dotted yellow lines represent *step distance* between nerve fibers. **B** A DICOM slide of nanoCT dataset. **C** Confirming histological slide at the corresponding specimen level with nerve fibers marked with green. Blue crosses represent the same anatomical structure and level for Fig. **B** and **C**. *SMAP* superior mesenteric artery plexus (Color figure online)

**Fig. 8:**
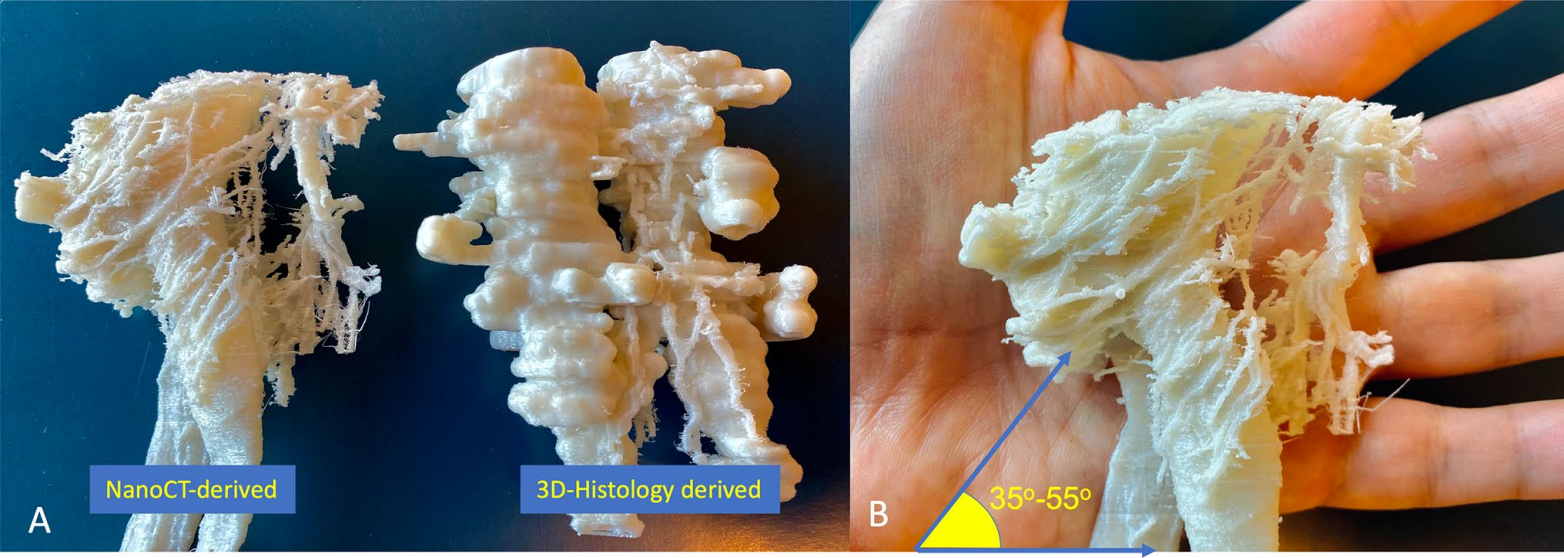
3D-printed models. **A** Physical models of the nanoCT-derived and 3D histology-derived SMAP in relation with the superior mesenteric artery and vein. **B** 3D-printed model 1:2 ratio illustrating the SMAP inclination angle range between 35° and 55°

3D histology reconstructions were produced from an average of 80 histological slides per specimen by segmenting nerves with a diameter between 300 and 800 um. The trajectory and position of these nerves along mesenteric vessels are presented in Fig. 6. NanoCT 3D reconstructions demonstrate several anatomical structures, including the superior mesenteric artery and vein (flattened out), and the network of nerve fibers representing the SMA plexus. Collagenous fibers, lymph vessels, and lymph nodes were omitted from the reconstruction to emphasize the nerve architecture. There are no SMA plexus fibers that were observed in the close periphery of the SMV. See Fig. 7. The nanoCT-derived 3D model shows the SMA plexus architecture having an oblique trajectory along the SMA in a counterclockwise direction (superior to inferior and medial to lateral direction). This can be better visualized on Fig. 8A through the printed version of this 3D model. Overall single nerve fibers encircle the outermost layer of the SMA with inclination angles between 35° and 55° from the transverse axis of the D3 volume (Fig. 8B). The average *step distance* of 2.45 mm is measured between each nerve fiber inclination as shown in Fig. 7A. Nerve fibers on the nanoCT 3D model are later confirmed histologically on the matching slicing level of the specimen, see Fig. 7B and C.

## Discussion

A review of the literature shows that a clear definition of the SMA vascular sheath is still missing [17, 25–30]. The results of anatomical dissection in this study reveal only one surgical dissection plane. This plane separates the SMA adventitia from a paravascular sheath as seen on our histological slides (Fig. 4). In this way, it separates the contents of the SMA adventitia (lymphatics, intrinsic nerves, and vasa vasorum) from those of the paravascular SMA sheath, which in turn supply the mesentery and the gut [18, 31]. Moreover, the SMA paravascular sheath thickness (Group I and II results) decreases caudally [32], still allowing the identification of this plane, consequently maximizing the extent of lymphadenectomy.

With the current trend to increase the extent of mesenterectomy in cancer surgery [33–35], concern has been raised about postoperative bowel function in patients where the SMAP may have been injured [8, 36]. It is also significant to point out that the extent of this injury depends not only on the morphology of the plexus but also on the surgical technique deployed. The results of this study clearly show that the SMAP is inherent to the paravascular sheath of the SMA, and not in the periphery of the SMV. These results also represent the first attempt to define the extent of SMAP injury in the context of a clinical trial, following a clearly defined surgical technique for performing right colectomy for cancer [16, 37]. This procedure involves a more radical (extended) D3 mesenterectomy than the *traditional* D3 right colectomy, namely operating within the arterial sheath of the SMA. The results of this study are therefore not transferable to the traditional mesenterectomy where these nerves are not damaged. It is also relevant to point out that this nerve plexus analysis has been performed at the most cranial level of the D3 dissection (5 mm proximal to the origin of the middle colic artery and where the potential for injury to the plexus is maximized), using a representative histopathological slide to establish nerve number and surface area removed. This singular slide actually represents the *transectional* injury to the SMAP, where our comparisons reveal a considerable injury (59% nerve fibers and 49% nerve area). Analysis of histological slides also lets us conclude that a relatively fewer number of nerve fibers, although larger in diameter, are left behind in the patient.

A literature search on postoperative intractable diarrhea after SMAP injury identified seven relevant articles [1, 2, 7, 32, 38–40]. The great majority of these publications concern surgery for pancreatic cancer and entail an *excisional* injury to the SMA paravascular sheath, including nerve ganglions [2, 41], between the origin of the SMA and the inferior border of the pancreas. Needless to say, the pancreatic head or the whole pancreas has been removed. Furthermore, the disruption of the bowel continuity during pancreas surgery also transects the bowel’s intrinsic nerve plexus, implying intrinsic denervation. These articles are not randomized controlled trials and contain small numbers of patients, rendering the level of the evidence low. A newer publication on the subject [5] compares two groups of 37 patients operated for pancreatic cancer in a randomized setting where the intervention was either right half dissection of the SMAP or SMAP preservation. The results seem to imply that diarrhea was more often present in the right half dissection of the SMAP (70.3%) when compared to preservation (48.6%), but still not significant. Perhaps more important is the fact that almost 60% of all these patients had recurrence. A previous study by Inoue et al. [41] also presents similar results.

We postulate that the oblique trajectory of the SMAP nerves demonstrated here leads to a more extensive, if not complete, injury due to a longitudinal excision of the SMAP performed at surgery. The potential level of damage to the SMAP, along the border of the SMA, is even more clear while observing the 3D-printed model on Fig. 8a. It is also worth noting that it was easier to measure SMAP trajectory angle with the physical 3D-printed models [42]. The functional outcome after the extended D3 mesenterectomy has been investigated and described in previous articles by Thorsen et al. When referring to the small bowel motility in these articles, it is essential to point out that the nerve injury caused by this specific surgical procedure exclusively involves the extrinsic nerves (SMAP), not the intrinsic (Enteric Nervous System), since the small bowel continuity is preserved. The amount of nerve injury described in this study can explain the “rapid small bowel transit” shown in Thorsen’s motility study performed three weeks after surgery [13]. More specifically, it manifests through a short period with frequent, loose stools with a maximum point 9 days after the operation. This stool pattern differs significantly from the stool pattern in patients, undergoing right colectomy with a traditional mesenterectomy without nerve damage [43]. Despite the extensive damage, a fast normalization process begins shortly after surgery, probably reflecting the bowel's extraordinary plasticity. A slow normalization over months follows. Six months after the operation, the small bowel transit time shows a clear tendency toward normalization [13]. In the long-term follow-up, the patients who have undergone the extended D3 mesenterectomy still present a slightly increased stool frequency and reduced consistency compared to those who have undergone the traditional mesenterectomy. However, this does not affect the long-term quality-of-life scores, which are equal in both groups [9, 14, 44].

The previously described interarterial nerve fibers between branches of the SMA [29], adaptation of the remaining nerves, as well as reinnervation [45] can provide an explanation for the normalization of postoperative bowel function that occurs during the first two years after surgery. Moreover, Thorsen et al. [14] have demonstrated that individual anatomical traits in patients, such as branching patterns of jejunal arteries, can have an impact on bowel function after surgery. Namely, having no jejunal artery proximal to the origin of the middle colic artery led to more diarrhea in patients operated with right colectomy and extended D3 mesenterectomy. In comparison with SMAP injury in pancreas surgery, the level of SMAP injury, while performing right colectomy with extended D3 mesenterectomy, lies distal to the inferior border of the pancreas and stretches to 1 cm below the origin of the ICA. With right colectomy, neither ganglions nor pancreatic tissue are removed, providing another possible explanation for the difference in clinical presentation between the two procedures.

The strength of this article is the multimethodological, macro/micro, 2D/3D, destructive/nondestructive approaches that allowed us to perform a composite interpretation. Characterizing the architecture of the SMAP was achieved using different technologies, namely 3D histology, nanoCT reconstructions, 3D printing, and supplemented with traditional anatomical dissection. While anatomical dissection showed nerve patterns and limits within the D3 area, histological imaging allowed for nerve count and area quantification. NanoCT imaging showed plexus trajectory and angulation. For these reasons, we deem it as important to combine both destructive and nondestructive imaging techniques to assure accurate anatomical information in future investigations. The number of anatomical (12) and clinical (9) specimens can be deemed sufficient when compared to other similar articles. Methodologically, it is important to remark that accurate measurements can be affected by different degrees of tissue shrinkage after the use of different fixation or contrast media. While the extension of shrinkage varies according to tissue properties, one must also consider a degree of tissue retraction after surgical excision (prior to fixation). Overall, we can generalize that our specimens retracted between 4 and 6% in all dimensions after excision and fixation [46].

When 3D histological reconstructions are in concern, a weakness is found in the manual alignment process [47]. The true trajectory of nerve fibers, namely their oblique direction along the SMA, was first noted on nanoCT scans, after 3D histology models had already been reconstructed. This can be explained by the fact that 3D histology reconstruction involves manual alignment and segmentation steps, both time-consuming and *user-dependent* processes.

## Conclusion

This article documents how the SMAP network is contained within the paravascular sheath of the SMA. The extent of injury caused to the SMAP, while performing right colectomy with extended D3 mesenterectomy, was 48% in nerve area and 59% in nerve count. This knowledge, in turn, provides a better insight into the differences between *excision* and *transection* injury of the SMAP. Additionally, the 35°–55° inclination range of the SMAP fibers can imply an even larger injury of the plexus when a longitudinal excision is performed. Finally, the implementation of multiple research methodologies allowed for a unique and comprehensive understanding of plexus topography and microanatomy.
